# Protection of Tong-Sai-Mai Decoction against Apoptosis Induced by H_2_O_2_ in PC12 Cells: Mechanisms via Bcl-2-Mitochondria-ROS-INOS Pathway

**DOI:** 10.1155/2014/371419

**Published:** 2014-10-22

**Authors:** Maxwell Kim Kit Lee, Yin Lu, Liu-qing Di, Hui-qin Xu

**Affiliations:** ^1^Jiangsu Engineering Research Center for Efficient Delivery System of Traditional Chinese Medicine, Nanjing 210046, China; ^2^Jiangsu Province Key Laboratory for Pharmacology and Safety Evaluation of Chinese Materia Medica, Nanjing, China; ^3^School of Pharmacy, Nanjing University of Chinese Medicine, 138 Xianlin Street, Nanjing 210046, China

## Abstract

Tong-Sai-Mai decoction (TSM) is a Chinese materia medica polyherbal formulation that has been applied in treating brain ischemia for hundreds of years. Because it could repress the oxidative stress in in vivo studies, now we focus on the in vitro studies to investigate the mechanism by targeting the oxidative stress dependent signaling. The relation between the neurogenesis and the reactive oxygen species (ROS) production remains largely unexamined. PC12 cells are excitable cell types widely used as in vitro model for neuronal cells. Most marker genes that are related to neurotoxicity, apoptosis, and cell cycles are expressed at high levels in these cells. The aim of the present study is to explore the cytoprotection of TSM against hydrogen peroxide- (H_2_O_2_-) induced apoptosis and the molecular mechanisms underlying PC12 cells. Our findings revealed that TSM cotreatment with H_2_O_2_ restores the expression of bcl-2, inducible nitric oxide synthase (INOS), and mitochondria membrane potential. Meanwhile, it reduces intracellular [Ca^2+^] concentration, lactate dehydrogenase (LDH) release, and the expression of caspase-3 and bax. The results of the present study suggested that the cytoprotective effects of the TSM might be mediated, at least in part, by the bcl-2-mitochondria-ROS-INOS pathway. Due to its nontoxic characteristics, TSM could be further developed to treat the neurodegenerative diseases which are closely associated with the oxidative stress.

## 1. Introduction

The brain ischemia is the third lethal factor of the death after the heart disease and cancer [1]. It is characterized by acute fainting, unconsciousness excessive phlegm, hemiparesis, dysphasia, facial palsy, and motor disorders. In the recent years, there are some reports that have revealed that the ischemic preconditioning (IPC) has obviously performed the protective qualities in the heart [2], brain [3], skeletal muscle [4], kidney [5], endothelium [6], and others. For example, in the brain, a 2 min ischemia 1 or 2 days prior to the 5 min ischemic insult is capable of protecting against the neuronal death [7]. This concept has been expanded to the preconditioning triggered by the nonischemic stress like chemicals irritation [8], hypoxia [9], and the reactive oxygen radicals [10]. For example, Sharma and Singh [11] had indicated that the preconditioning with the oxidative stress may play cardioprotection role against the ischemia reperfusion injury. Another example like Lee et al. [12] studied that the oxidant (H_2_O_2_) preconditioning could protect the human proximal tubular cells against lethal oxidant injury.

In the recent years, the normal and diseased postnatal CNS oxidation state has become the tremendous interest subject for the study. The brain provides a highly oxidized environment that is normally vulnerable to the oxidative stress due to the brain's high oxygen consumption rate, its abundant lipid content, and the antioxidant enzymes relative paucity compared with the other tissues [13]. Within the CNS the balance of the oxidative stress between the generation and degradation of ROS is tightly controlled [14] and could disrupt the equilibrium and thus could be classified as a contributor to multiple diseases and participate in the neuronal damage. The free radicals such as H_2_O_2_, superoxide, and others would react with the membrane lipids, enzymes, and other essential cell components, resulting in the cell death. There are several reports that have demonstrated that ROS are involved in the several neurodegenerative diseases pathophysiology such as Alzheimer's diseases [15, 16], Parkinson's diseases [17], stroke [18], and ALS [19]. The oxidative stress is also thought to lead to dysfunction in otherwise normal tissue as a result of ionizing radiation therapy against brain tumors, particularly in the dividing cells [20, 21]. With the increasing relevance to a wide range of the diseases, identifying the oxidative stress has been a long-held target for pharmaceutical and therapeutic intervention.

On the other hand, there are some studies that have demonstrated that ROS can exert IPC-like protective effects in the ischemic/reperfusion myocardium [22, 23]. Recent reports also have shown that ROS can alter the mitochondrial function and the mitochondrial permeability transition pore [24, 25]. Proverbially, the apoptosis process is a process which involves changes in the expression of a distinct set of genes. One of the major genes in charge of regulating the apoptosis is the protooncogene bcl-2. The bcl-2 protein has been classified as an antiapoptotic protein [26]. There are several studies that have revealed that the bcl-2 could downregulate the various apoptotic stress induced apoptosis of the neuronal cells [27, 28]. On the contrary, the bcl-2 overexpression prevented it from the oxidant cellular insults or the calcium influx induced cell death in nonneuronal cells [29]. In addition, the bcl-2 overexpression afforded to protect against A*β*-induced apoptosis in PC12 cells as well as the primary cortical neurons [30, 31].

Furthermore, there is an accumulating evidence indicating that the inducible isoform of NO synthase (INOS) mediated the cardioprotective effects induced by the ischemic preconditioning (IPC) [32, 33]. If INOS activity was ablated by the pharmacological or the genetic approach, the protective effects of late PC were abrogated [34, 35]. Apart from that, INOS also plays a crucial role in the delayed cardioprotection induced by the various pharmacological agents including adenosine A1 receptor agonists [35–37] and opioid 1 receptor agonists [36, 37], implying that different stimuli could elicit the INOS in the cardioprotection [32, 33]. Hence, there are some researchers who have not proved a protective role of INOS during the late PC [38].

Despite the fact that the extensive interest had focused on the chemical medicine and ischemic preconditioning in the brain ischemia treatment, in the present study we sought that the traditional herbal medicine could treat and prevent the brain ischemia. Tong-Sai-Mai decoction, which is a traditional polyherbal formulation Chinese medicine, widely applied in treating the neurodegenerative diseases like the brain stroke. It shows a variety of pharmacological effects, including treating the thromboangiitis obliterans [39, 40], arteriosclerotic obliteration [41], brain stroke [42, 43], coronary heart disease [44], and others. However, whether TSM could provide protection against apoptosis induced by hydrogen peroxide, H_2_O_2_, is still unknown.

In the present study, we examined the hypothesis that TSM affords protection against H_2_O_2_-induced PC12 cells apoptosis by bcl-2- (an antiapoptotic protein-) mitochondria-ROS-INOS pathway, to investigate whether TSM could protect PC12 cells by the repetitive exposure to H_2_O_2_ against the apoptotic consequences of the subsequent oxidative insults and whether the H_2_O_2_-induced TSM adaptive cytoprotection related to the changes in mitochondrial membrane potential, ROS, INOS, and the bcl-2 expressions so as to explore a suitable candidate for neuroprotective therapy for the brain ischemia.

## 2. Methods and Materials

### 2.1. Preparation of TSM

All the crude drugs including 231.5 g* Astragalus membranaceus*, 231.5 g Radix Scrophulariae, 231.5 g licorice, 231.5 g* Dendrobium nobile*, 231.5 g* Angelica sinensis*, 231.5 g honeysuckle, and 231.5 g Radix Achyranthis Bidentatae were purchased from Jiangsu Provincial Hospital of Traditional Chinese Medicine (Jiangsu Province, China). The voucher sample (number 120320) has been deposited in the College of Pharmacy, Nanjing Chinese Medicine University. Firstly, the mixture of TSM was soaked in the 70% ethanol (1 : 6; w/v) for 0.5 h at the room temperature and prepared by boiling for 1.5 h. The cooled decoction was filtered through two layers of cotton gauze. Then, the residue was soaked again in the 70% ethanol (1 : 4; w/v) for 0.5 h at the room temperature and prepared by boiling for 1.5 h. The cooled decoction was then filtered through the two layers of cotton gauze. Finally, the residue was soaked again in the 50% ethanol (1 : 4; w/v) for 0.5 h at the room temperature and prepared by boiling for 0.5 h. The cooled decoction was then filtered through the two layers of cotton gauze. The solution obtained was concentrated into the residues in a vacuum evaporator and lyophilized into the powder. The yield of TSM extract was 18.23 (w/w) according to the original herbs.

### 2.2. Materials

Hydrogen peroxide (H_2_O_2_) (number 070610) was purchased from Nanjing Chemical Reagent Factory. RPMI-1640 medium, fetal bovine serum, 0.5% trypsin EDTA, penicillin, and streptomycin were purchased from Gibco (Grand Island, NY, USA). LDH diagnostic kit was purchased from Stanbio Laboratory, USA. Hoechst 33258, JC-1, fura-2/AM, ROS, and INOS were purchased from Beyontime. Propidium iodide (PI) was purchased from Sigma Chemical Co. Ltd. (St. Louis, MO, USA). The specific monoclonal anti-bcl-2 antibody, anti-bax antibody, and anti-caspase-3 antibody were obtained from Cell Signaling Int. All other chemicals and reagents were of analytical grade.

### 2.3. Cell Culture and Preconditioning Protocols

The PC12 cells, a rat cell line derived from pheochromocytoma cells, were supplied from Shanghai Institutes for Biological Sciences, Chinese Academy of Cell Resource Center, and were maintained on tissue culture plastic in RPMI-1640 medium supplemented with 10% heat-inactivated horse serum and 5% fetal bovine serum (FBS) at 37°C under the atmospheric pressure of 5% CO_2_ and 95% air. The culture media were changed three times per week. To study the neuroprotective effect of the Tong-Sai-Mai decoction, PC12 cells were divided into five equal groups: nontreated control, 200 *μ*M hydrogen peroxide (H_2_O_2_), and 200 *μ*M hydrogen peroxide (H_2_O_2_) plus with Tong-Sai-Mai decoction  (0.05 mg/mL, 0.5 mg/mL, and 5 mg/mL) in all the experiments. Experiments were carried out 24 h after the cells were seeded. After the media were removed, the cells were cultured in serum-free medium and incubated with the corresponding drugs for another 24 h.

### 2.4. Determination of Cell Viability

PC12 cells were plated at a density of 1 × 10^4^ cells/well in 96-well plates and the cell viability was determined by the conventional 3-(4,5-dimethylthiazol-2-yl)-2,5-diphenyltetrazolium bromide (MTT) reduction assay. The MTT assay relied primarily on the mitochondrial metabolic capacity of viable cells and reflected the intracellular redox state. After the incubation process, the cells were treated with the MTT (Sigma, USA) solution (final concentration, 0.5 mg/mL) for 4 hours. The medium was removed and 150 *μ*L of dimethyl sulfoxide (DMSO) was added into each well. The formazan dye at 490 nm was measured with a microplate reader (Molecular Devices, Sunnyvale, CA, USA). The results were expressed as the percentage of MTT reduction rate, assuming that the absorbance of the control cells was 100%.

### 2.5. Lactate Dehydrogenase (LDH) Assay

The LDH activity was measured by using a LDH diagnostic kit (Stanbio Laboratory, USA) according to the manufacturer's protocol. Briefly, the PC12 cells were seeded on 24-well culture plates at a density of 1 × 10^5^ cells/well. At the end of the treatment, the medium was collected to measure the LDH activity. The absorbance of the samples was recorded at 340 nm at 1 min intervals for 3 min. The change in absorbance was then expressed in concentration units per liter. To determine the intracellular LDH activity, the cells were washed by D-Hanks and then 500 *μ*L D-Hanks were added into each well. The cells were lysed by ultrasonication (750 W) for 30 s. The suspension was centrifuged and the supernatants were collected for assay of the LDH activity. The LDH leakage was expressed as the percentage (%) of the total LDH activity (LDH in the medium + LDH in the cell), according to the equation % LDH released = (LDH activity in the medium/total LDH activity) × 100.

### 2.6. Quantification of DNA Fragmentation

The quantification of DNA fragmentation was determined by Cell Death ELISA Kit (Roche Applied Sciences, Basel, Switzerland) according to the manufacturer's protocol. Briefly, PC12 cells were seeded on a 96-well culture plate at a density of 2 × 10^4^ cells/well. At the end of the treatment, the cells were resuspended and incubated in 200 *μ*L of lysis buffer for 30 min at the room temperature. After being centrifuged to remove nuclei and cellular debris, 20 *μ*L of the supernatant of each sample was transferred to a streptavidin-coated microplate and incubated for 2 h at the room temperature with an immunoreagent containing two monoclonal antibodies, anti-histone (biotin-labelled) and anti-DNA (peroxidase-conjugated). After being washed three times with incubation buffer, each well was treated with the chromogen 2,2′-azinobis(3-ethylbinzthiazoline-6-sulfonic acid) as a substrate and then incubated for 15 min. The intensity of the color that developed was measured at 405 nm, while that at 490 nm was used as a blank (reference wavelength). The results were expressed as percentage of nontreated control.

### 2.7. Measurement of Intracellular Ca^2+^ Concentration

The concentration of [Ca^2+^]_*i*_ was determined as described previously elsewhere [45, 46]. Briefly, PC12 cells were seeded on 24-well plates at a density of 1 × 10^5^ cells/well. At the end of the treatment, the cells were collected and incubated with the complete medium containing 5 *μ* M fura-2/AM at 37°C for 45 min. Subsequently, the cells were washed and resuspended with cold balanced salt solution buffer containing 0.2% bovine serum albumin. The cells were incubated at 37°C for another 5 min just prior to the measurement. The concentration of [Ca^2+^]_*i*_ was determined by alternating excitation wavelengths between 340 nm and 380 nm with emission at 510 nm by using the fluorescence spectrophotometer (F-4500, HITACHI, Japan). The results were expressed as the percentage of the nontreated control.

### 2.8. The Nuclear Staining for Assessment of Apoptosis

The chromosomal condensation and morphological changes in the nucleus of PC12 cells were observed by using the chromatin dye Hoechst 33258. The PC12 cells were fixed with 4% paraformaldehyde in 0.1 M phosphate buffered saline (PBS) for 10 min. After three rinses with PBS solution, the cells were stained with 5 mg/mL Hoechst 33258 for 10 min. The slides were rinsed briefly with PBS, air-dried, then mounted in an antifluorescein fading medium (Perma Fluor, Immunon, PA, USA). The slides were visualized under a fluorescent microscope (BX50-FLA, Olympus, Tokyo, Japan). The viable cells displayed normal nuclear size and uniform fluorescence, whereas the apoptotic indicated the condensed nuclei or nuclear condensations. The percentage of apoptotic cells was evaluated as follows: the percentage of apoptotic cells = the number of apoptotic cells/(the number of apoptotic cells + the number of viable cells) × 100%.

### 2.9. The Annexin V/PI Analysis for Cell Apoptosis

The apoptotic cells were determined by the annexin V/PI analysis according to the manufacturer's protocol. Briefly, the PC12 cells were seeded on the 6-well plates at a density of 2 × 10^4^ cells/well. At the end of the treatment, the treated PC12 cells were digested with trypsin (2.5 g/L) and centrifuged at 1500 rpm for 10 min; the supernatant aliquot was removed. The apoptosis in cells was induced using the desired method. A negative control was prepared by incubating cells in the absence of inducing agent. The cells were harvested after the incubation period and washed in the cold phosphate buffered saline (PBS). Then 1X annexin-binding buffer was prepared. The 100 *μ*g/mL working of PI was prepared by diluting 5 *μ*L of the 1 mg/mL PI stock solution (component B) in 45 *μ*L 1X annexin-binding buffer. The unused portion of this working solution was stored for future experiments. The washed cells were recentrifuged, the supernatant was discarded, and the cells were resuspended in 1X annexin-binding buffer. The cell density was determined and diluted in the 1X annexin-binding buffer to ~1 × 10^6^ cells/mL, preparing a sufficient volume to have 100 *μ*L per assay. The 5 *μ*L Alexa Fluor 488 annexin V (component A) and 1 *μ*L 100 *μ*g/mL PI working solution were added to each 100 *μ*L of cell suspension. After that, the cells were incubated at room temperature for 15 minutes. After the incubation period, the 400 *μ*L 1X annexin-binding buffer was added and mixed gently and the sample was kept on ice. As soon as possible, the stained cells were analysed by flow cytometry, measuring the fluorescence emission at 530 nm and 570 nm.

### 2.10. Analysis of Cell Cycle Distribution

The effect of TSM on the DNA content was performed by the cell cycle phase distribution analysis in PC12 cells. Briefly, the cells were harvested after treatment with TSM for 24 h, followed by fixing with ice-cold 70% ethanol, and stored at −20°C overnight. The cells were resuspended in 100 *μ*L RNase (10 mg/mL) at 37°C for 30 min and stained with PI (50 *μ*g/mL) at 4°C in the dark for 30 min. The cells were detected by the flow cytometer (FACS Calbur Becton-Dickinson, USA).

### 2.11. Measurement of the Mitochondrial Membrane Potential (MMP)

JC-1 is a widely used ideal fluorescent probe for detecting ΔΨ M and can be used for mitochondrial membrane potential detection cell. When the mitochondrial membrane potential is high, forming polymer accumulation of JC-1 in the matrix of mitochondria and can emit red fluorescence; when the mitochondrial membrane potential is low, JC-1 cannot gather in the matrix of mitochondria, and when JC-1 is a monomer, it can produce a green fluorescence. Green and red fluorescence compared with commonly used to measure the mitochondrial depolarization. PC12 cells were incubated in 6-well plates after drug treatment for 24 hours, the culture medium was removed, the addition of an equal volume of JC-1 (5 mg/L) was added and incubated for 20 min at 37°C for cell culture, washed 2 times with PBS. Laser scanning confocal microscope (Zeiss), with the excitation wavelength of 488 nm, emission of green and red fluorescence intensity wavelength 545 nm, was used for detection. The change to green and red fluorescence intensity ratio reflected the mitochondrial membrane potential.

Next, we also measure the mitochondrial membrane potential (MMP) to be monitored by using a flow cytometer (BECKMAN-COULTER Co., USA) and the fluorescent dye Rh 123, a cell permeable cationic dye, which preferentially enters into the mitochondria based on the highly negative MMP. The depolarization of MMP results in the loss of Rh 123 from the mitochondria and a decrease in the intracellular fluorescence. The Rh 123 (100 *μ*g/L) was added to the cell cultures for 45 min at 37°C. The cells were collected by pipetting and washed twice with PBS. Ten thousand cells per sample were analyzed by the flow cytometry.

### 2.12. Measurement of Intracellular ROS Generation

The intracellular ROS were determined by the oxidative conversion of cell permeable 2′,7′-dichlorofluorescein diacetate (DCFA-DA) to fluorescent 2′,7′-dichlorofluorescein (DCF) [47, 48]. The cells were collected by pipetting and were washed once time with the PBS. After DCFH-DA (2.5 *μ* M) was added to cell cultures for 20 min at 37°C, the cells were washed twice with PBS. The DCF fluorescence was measured over the whole field of vision by using a fluorescent microscope connected to an imaging system (BX50-FLA, Olympus, Tokyo, Japan). In addition, the mean fluorescent intensity (MFI) of the positive cells in ten thousand cells per sample was measured by FCM, and the MFI represents the amount of ROS accumulation.

### 2.13. Western Blot Analysis for Bcl-2, Bax, and Caspase-3 Expressions

The SDS-polyacrylamide gel electrophoresis (PAGE) was carried out on 5% stacking and 12% resolving gel with low range molecular weight standards (Solarbio, China). Equal amounts of proteins were loaded in each lane with loading buffer (Beyotime, China) containing 0.1 M Tris (pH 6.8), 20% glycerol, 10% mercaptoethanol, 4% SDS, and 0.2% Bromophenol Blue. Samples were heated at 100°C for 5 min before gel loading. Following the electrophoresis, the proteins were transferred to a PVDF transfer membrane (Solarbio, China). After this, the membranes were blocked with TBST (50 m M Tris-HCl, pH 7.4, 0.15 M NaCl, and 0.1% Tween-20) containing 5% BSA (Sigma, USA) for 2 h. Following this, the membranes were incubated with rats monoclonal anti-bcl-2, anti-bax, and anti-caspase-3 primary antibodies (Cell Signaling Technology, USA) diluted at 1 : 1000 at 4°C overnight. After washing with TBST, the membranes were incubated with anti-rat Ig G labeled with horseradish peroxidase (Zsbio, China) diluted at 1 : 1000 at room temperature for 2 h. The membranes were washed again and developed with an enhanced chemiluminescence system (ECL, Zsbio, China) followed by apposition of the membranes with autoradiographic films (Kodak, China). The integrated optical density for the protein band was calculated by Image-J-software.

### 2.14. Measurement of INOS Expression

The cells were collected by pipetting and adjusted to a concentration of 1 × 10^7^ cells/well. The cells were then incubated with specific monoclonal anti-INOS antibody at room temperature and washed twice with PBS containing 10% fetal bovine serum. The cells were incubated again with goat anti-rat Ig G-FITC (1 : 200) diluted by 0.5% fetal bovine serum/PBS for 30 min at room temperature. Washed twice with PBS, ten thousand cells per sample were analyzed by flow cytometer; the mean fluorescence intensity (MFI) in the positive cells represents percentage of INOS expression.

### 2.15. Statistical Analysis

Data are expressed as mean ± SEM. Differences among groups were tested by one-way ANOVA. The comparisons between the two groups were performed by an unpaired Student's *t*-test. A value of *P* < 0.05 was considered statistically different.

## 3. Results

### 3.1. TSM Protects PC12 Cells against H_2_O_2_-Induced Cytotoxicity

**Figure 1:**
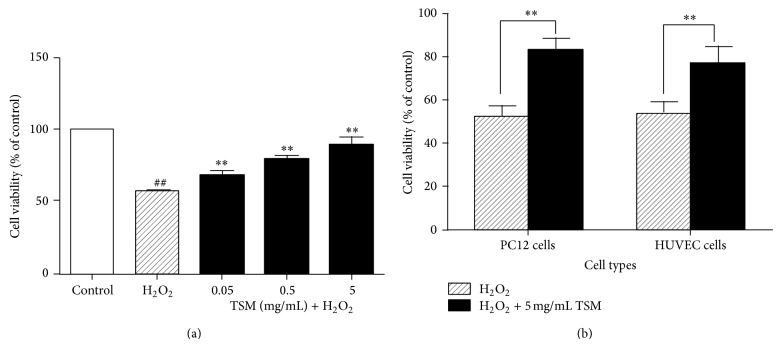
(a) The protective effect of TSM on the cell viability in H_2_O_2_-treated PC12 cells. The cells were treated with 200 *μ*mol/L H_2_O_2_ and 0.05, 0.5, and 5 mg/mL TSM with 200 *μ*mol/L H_2_O_2_. The values given are the mean ± SEM (*n* = 6). ^#^ *P* ≤ 0.05 and ^##^ *P* ≤ 0.01 as compared with the control group; ^*^ *P* ≤ 0.05 and ^**^ *P* ≤ 0.01 as compared with the H_2_O_2_ group. (b) The effects of cell viability of H_2_O_2_ and H_2_O_2_ + 5 mg/mL TSM treated PC12 cells and HUVEC cells. The cells were treated with 200 *μ*mol/L H_2_O_2_ and (200 *μ*mol/L H_2_O_2_ + 5 mg/mL TSM) in the PC12 cells and HUVEC cells. The values given are the mean ± SEM (*n* = 6). ^##^ *P* ≤ 0.01 as compared with the control group.

The PC12 cells were incubated with different concentrations (0.05 mg/mL, 0.5 mg/mL, and 5 mg/mL) of TSM and H_2_O_2_, respectively, for 24 h and the cell viability was evaluated by determining the percentage of MTT reduction. The effect of TSM on the cell viability in H_2_O_2_-treated PC12 cells was shown in Figure 1(a). The viability of PC12 cells when exposed to H_2_O_2_ at the concentration of 200 *μ*mol/L for 24 h was significantly decreased as compared with the control group and the survival rate was 57.44% of control. When PC12 cells were treated with various concentrations of TSM (0.05 mg/mL, 0.5 mg/mL, and 5 mg/mL) in the presence of 200 *μ*mol/L H_2_O_2_ for 24 h, the cell viability was significantly increased as compared with the H_2_O_2_ group, and the survival rate was 68.84%, 79.18%, and 89.95% of control, respectively.

Next, in order to prove that the cotreatment of TSM can restore the H_2_O_2_ effect and the effect was not cell type specific, we also conducted with different types of cells treated with 200 *μ*mol/L H_2_O_2_ and (200 *μ*mol/L H_2_O_2_ + 5 mg/mL TSM) treated in PC12 cells and HUVEC cells determined by the MTT assay. The effect of cell viability of H_2_O_2_ and H_2_O_2_ + 5 mg/mL TSM treated PC12 cells and HUVEC cells was shown in Figure 1(b). When the PC12 cells were exposed to H_2_O_2_ at the concentration of 200 *μ*mol/L for 24 h, the cell viability was significantly decreased and the survival rate was 52.04%.  When the HUVEC cells were exposed to H_2_O_2_ at the concentration of 200 *μ*mol/L for 24 h, the cell viability was significantly reduced and the survival rate was 54%. When the PC12 cells were cotreated by the 200 *μ*mol/L H_2_O_2_ + 5 mg/mL TSM, the cell viability was significantly increased as compared with the H_2_O_2_ group and the survival rate was 83.5%. When the HUVEC cells were cotreated by the 200 *μ*mol/L H_2_O_2_ + 5 mg/mL TSM, the cell viability was significantly increased as compared with the H_2_O_2_ group and the survival rate was 76.74%. Therefore, the cotreatment of TSM can restore the H_2_O_2_ effect not only in PC12 cells, but also in HUVEC cells.

### 3.2. Effect of TSM on the LDH Assay in H_2_O_2_-Treated PC12 Cells

**Figure 2:**
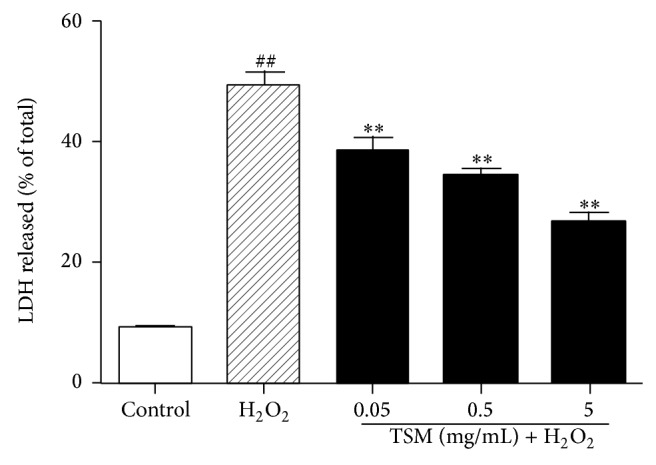
Effect of TSM on the LDH leakage in H_2_O_2_-treated PC12 cells. The cells were treated with 200 *μ*mol/L H_2_O_2_ and 0.05, 0.5, and 5 mg/mL TSM with 200 *μ*mol/L H_2_O_2_. The values given are the mean ± SEM (*n* = 6). ^#^ *P* ≤ 0.05 and ^##^ *P* ≤ 0.01 as compared with the control group; ^*^ *P* ≤ 0.05 and ^**^ *P* ≤ 0.01 as compared with the H_2_O_2_ group.

The effect of the TSM on the LDH assay in H_2_O_2_-treated PC12 cells was shown in Figure 2. After the treatment of PC12 cells with 200 *μ*mol/L H_2_O_2_ for 24 h, the LDH assay was significantly increased as compared with the control group. The LDH activity in H_2_O_2_ group was 49.5% while being 9.2% in control group. The cotreatment of PC12 cells with various concentrations of TSM (0.05 mg/mL, 0.5 mg/mL, and 5 mg/mL) in the presence of 200 *μ*mol/L H_2_O_2_ for 24 h resulted in decrease of LDH activity with 38.5%, 34.33%, and 26.5%, respectively.

### 3.3. Effect of TSM on the [Ca^2+^]_*i*_ Concentration in H_2_O_2_-Treated PC12 Cells

**Figure 3:**
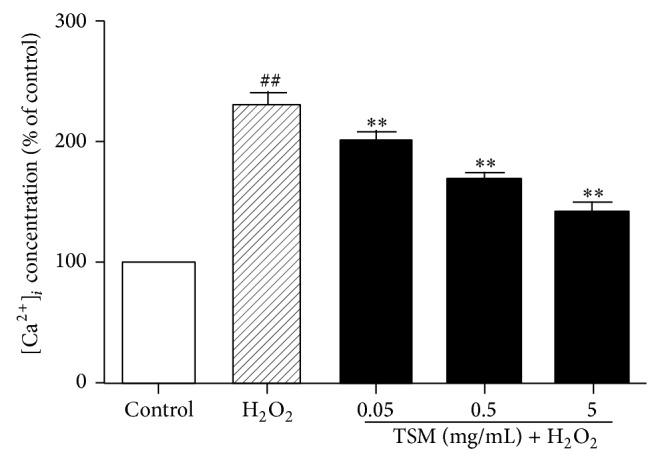
Effect of TSM on the [Ca^2+^]_*i*_ concentration in H_2_O_2_-treated PC12 cells. The cells were treated with 200 *μ*mol/L H_2_O_2_ and 0.05, 0.5, and 5 mg/mL TSM with 200 *μ*mol/L H_2_O_2_. The values given are the mean ± SEM (*n* = 6). ^#^ *P* ≤ 0.05 and ^##^ *P* ≤ 0.01 as compared with the control group; ^*^ *P* ≤ 0.05 and ^**^ *P* ≤ 0.01 as compared with the H_2_O_2_ group.

The effect of TSM on the [Ca^2+^]_*i*_ concentration in H_2_O_2_-treated PC12 cells was indicated in Figure 3. After treatment of PC12 cells with 200 *μ*mol/L H_2_O_2_ for 24 h, the [Ca^2+^]_*i*_ concentration was significantly increased as compared with the control group and the [Ca^2+^]_*i*_ concentration 232.33% while that of 100% in the control group. When the PC12 cells were cotreated with various concentrations of TSM (0.05 mg/mL, 0.5 mg/mL, and 5 mg/mL) in the presence of 200 *μ*mol/L H_2_O_2_ for 24 h, the [Ca^2+^]_*i*_ concentration was significantly decreased to 200.5%, 168%, and 140.17%, respectively, as compared with the H_2_O_2_ group.

### 3.4. Effect of TSM on the DNA Fragmentation in H_2_O_2_-Treated PC12 Cells

**Figure 4:**
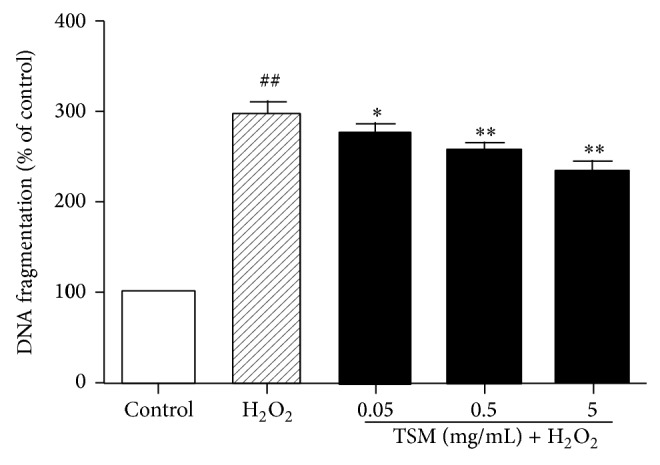
Effect of TSM on the DNA fragmentation in H_2_O_2_-treated PC12 cells. The cells were treated with 200 *μ*mol/L H_2_O_2_ and 0.05, 0.5, and 5 mg/mL TSM with 200 *μ*mol/L H_2_O_2_. The values given are the mean ± SEM (*n* = 6). ^#^ *P* ≤ 0.05 and ^##^ *P* ≤ 0.01 as compared with the control group; ^*^ *P* ≤ 0.05 and ^**^ *P* ≤ 0.01 as compared with the H_2_O_2_ group.

The effect of TSM on the DNA fragmentation in H_2_O_2_-treated PC12 cells was indicated in Figure 4. After treatment of PC12 cells with 200 *μ*mol/L H_2_O_2_ for 24 h, the DNA fragmentation was significantly increased as compared with the control group and the extent of DNA fragmentation was 301.33% while being 100% in the control group. When PC12 cells were cotreated with various concentrations of TSM (0.05 mg/mL, 0.5 mg/mL, and 5 mg/mL) in the presence of 200 *μ*mol/L H_2_O_2_ for 24 h, the DNA fragmentation was significantly reduced as compared with H_2_O_2_ group, and the extent of DNA fragmentation was 274.74%, 256.79%, and 235.73%, respectively.

### 3.5. TSM Protects PC12 Cells against H_2_O_2_-Induced Apoptosis

**Figure 5:**
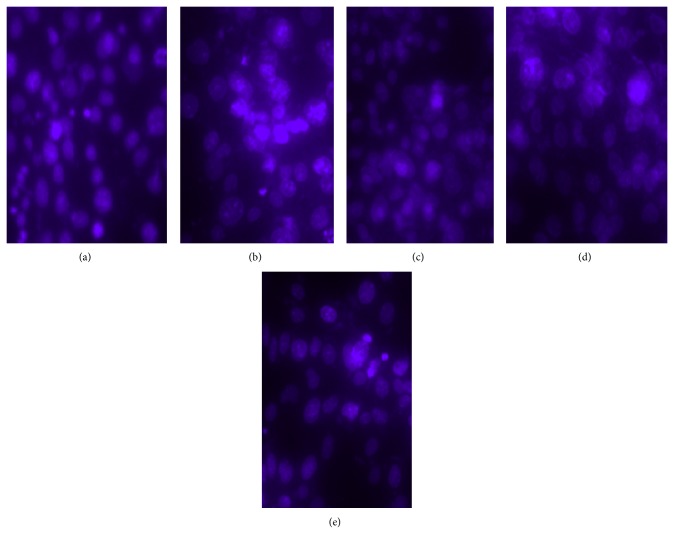
Nuclear staining to evaluate induction of PC12 cells apoptosis by H_2_O_2_ and the cytoprotection of TSM. The nuclear condensation was assessed by using Hoechst 33258 for PC12 cells. (a) Control group; (b) PC12 cells exposed to 200 *μ*mol/L H_2_O_2_ clearly showed the apoptotic nuclear condensation; ((c), (d), and (e)) PC12 cells, cotreated with 0.05, 0.5, and 5 mg/mL TSM with 200 *μ*mol/L H_2_O_2_, did not obviously exhibit such nuclear condensation.

The nuclear staining assay was applied to assess the morphological changes of apoptosis in PC12 cells. As demonstrated in Figure 5, the normal cells exhibited uniformly dispersed chromatin and intact cell membrane (Figure 5). The cells treated with 200 *μ*mol/L H_2_O_2_ for 24 h showed typical characteristics of apoptosis including the apoptotic nuclear condensation (Figure 5). However, in the cells which were cotreated with 0.05 mg/mL, 0.5 mg/mL, and 5 mg/mL TSM, respectively, and H_2_O_2_ at 200 *μ*mol/L for 24 h, the number of cells with nuclear condensation was significantly reduced (Figure 5), suggesting that TSM could protect PC12 cells against apoptosis induced by H_2_O_2_.

**Figure 6:**
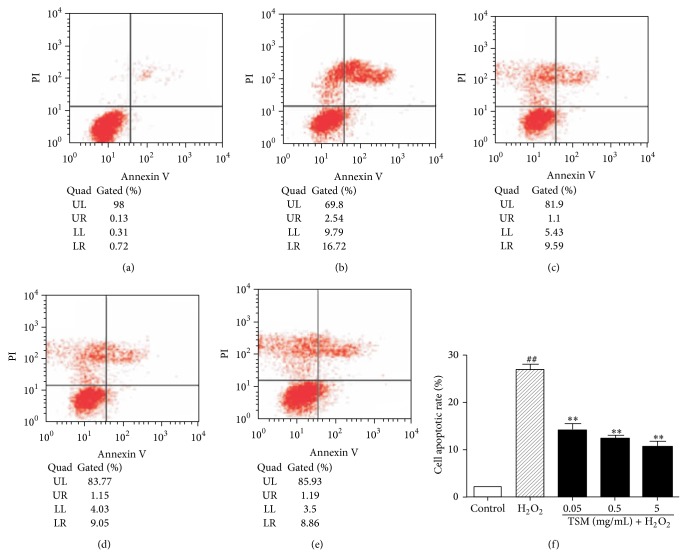
TSM protected PC12 cells against apoptosis induced by H_2_O_2_. (a) Control group. The cells were treated with 200 *μ*mol/L H_2_O_2_ (b) and 0.05, 0.5, and 5 mg/mL TSM in the presence of 200 *μ*mol/L H_2_O_2_ ((c), (d), and (e)). Annexin V+/PI− population (LR in a diagram) indicated early apoptosis and annexin V+/PI+ population (UR in diagram) indicated late apoptosis. The analysis was performed in triplicate and representative data was shown. The percentage of PC12 cells apoptosis was assessed by annexin V/PI FCM assay. ^#^ *P* ≤ 0.05 and ^##^ *P* ≤ 0.01 as compared with the control group; ^*^ *P* ≤ 0.05 and ^**^ *P* ≤ 0.01 as compared with the H_2_O_2_ group.

Similarly, the statistical findings from annexin V/PI analysis also revealed H_2_O_2_-induced PC12 cells apoptosis, shown in Figure 6. After the cells were treated with the indicated concentration 200 *μ*mol/L H_2_O_2_ for 24 h, the percentage of the apoptotic cells was enhanced to 27% (Figure 6). TSM had antiapoptotic effect. As shown in Figure 6, the cotreatment with 200 *μ*mol/L H_2_O_2_ and TSM at 0.05 mg/mL, 0.5 mg/mL, and 5 mg/mL reduced the percentage of the apoptotic cells to 14.1%, 12.23%, and 10.07%, respectively. Figure 6 demonstrated the annexin V/PI analysis on the protection of TSM at the indicated doses against the H_2_O_2_-induced apoptosis. All three concentrations of 0.05 mg/mL, 0.5 mg/mL, and 5 mg/mL TSM did not induce the cells apoptosis.

### 3.6. Cell Cycle Analysis by PI Staining

**Figure 7:**
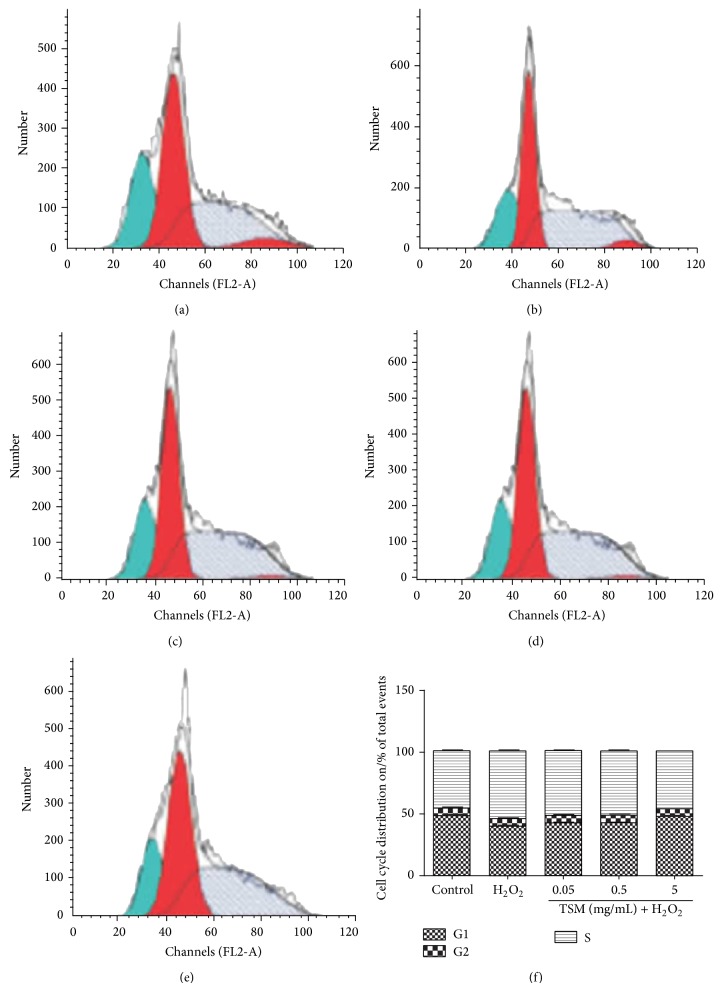
The cell cycle modifications induced by TSM. (a) Control group. The cells were treated with 200 *μ*mol/L H_2_O_2_ (b) and 0.05, 0.5, and 5 mg/mL TSM in the presence of 200 *μ*mol/L H_2_O_2_ ((c), (d), and (e)). The blue color represented the diploid G1 phase; the red color represented the diploid G2 phase; and the grey color represented the diploid S phase. The percentage of G1, G2, and S in cells treated with 200 *μ*mol/L H_2_O_2_ and various concentrations of TSM and cumulative data from three independent experiments were shown.

From the cell cycle analysis, we found that when the control group (G1 phase: 49%; G2 phase: 5%; S phase: 46%) was compared with the H_2_O_2_ group the cells at G1 phase reduced to 40.33% and the cells at S phase increased to 54.67%. After cotreatment with various concentrations of TSM and when compared with the H_2_O_2_ group, the G1 phase cells rate increased to 41.67% (0.05 mg/mL TSM group), 42.67% (0.5 mg/mL TSM group), and 47.67% (5 mg/mL TSM group), respectively; these three groups were higher than the H_2_O_2_ group. But for the S phase, the cells rate recovered to 53.33% (0.05 mg/mL TSM group), 52.33% (0.5 mg/mL TSM group), and 47.33% (5 mg/mL TSM group), lower than the H_2_O_2_ group, referring to Figure 7. The results revealed that in the H_2_O_2_ group the cell cycle was terminated at S phase; when adding the various concentrations of TSM we confirmed that it could attenuate this phenomenon.

### 3.7. TSM Prevents H_2_O_2_-Induced Dissipation of the Mitochondrial Membrane Potential (MMP)

**Figure 8:**
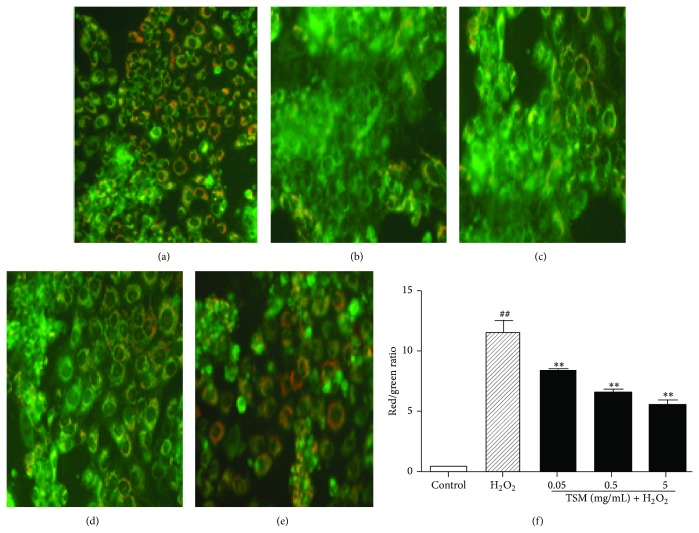
Revealing the cell ΔΨ M dissipation red/green ratio values induced by TSM. (a) Control group. The cells were treated with 200 *μ*mol/L H_2_O_2_ (b) and 0.05, 0.5, and 5 mg/mL TSM in the presence of 200 *μ*mol/L H_2_O_2_ ((c), (d), and (e)). Under normal mitochondria, the JC-1 accumulation in the mitochondrial matrix can form a red fluorescence polymer. Due to the decrease or loss of the mitochondrial membrane potential, JC-1 can exist in green fluorescence monomeric form. The mitochondria degree polarization can be measured by the ratio of red/green fluorescence intensity. The percentage of ΔΨ M dissipation in cells treated with 200 *μ*mol/L H_2_O_2_ and various concentrations of TSM and cumulative data from three independent experiments were shown.

**Figure 9:**
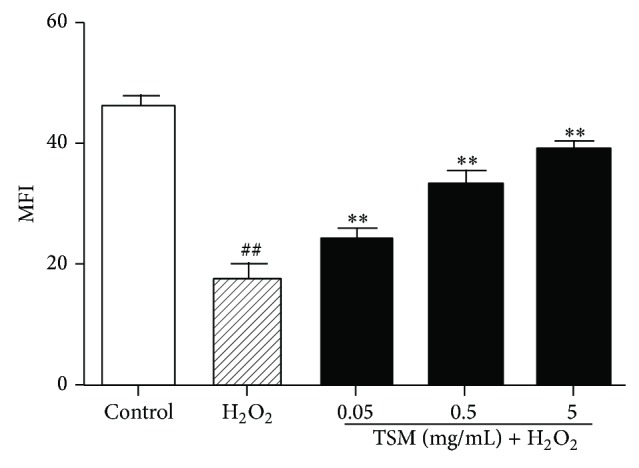
TSM blocked the H_2_O_2_-induced reduction of mitochondrial membrane potential (MMP). The MMP was assessed by the mean fluorescent intensity (MFI) of rhodamine 123 staining in ten thousand of PC12 cells. TSM with 0.05 mg/mL, 0.5 mg/mL, and 5 mg/mL, respectively, did not reduce the MMP, but partly blocked the MMP reduction of PC12 cells induced by 200 *μ*mol/L H_2_O_2_. ^#^ *P* ≤ 0.05 and ^##^ *P* ≤ 0.01 as compared with the control group; ^*^ *P* ≤ 0.05 and ^**^ *P* ≤ 0.01 as compared with the H_2_O_2_ group.

The dissipation of MMP is a critical event in the process of apoptosis [49]. To examine preservation of MMP involve in the antiapoptotic effect of TSM. Firstly, we conducted the fluorescence microscope (Figure 8) to study the cell ΔΨ MMP dissipation. From the results, we found that when the control group (the red/green ratio value = 0.3733) was compared with the H_2_O_2_ group, the red/green ratio value increased to 11.3 and there was a significant difference (*P* ≤ 0.01). After cotreatment with various concentrations of TSM and 200 *μ*mol/L H_2_O_2_ for 24 h and compared with the H_2_O_2_ group, we found that the red/green ratio values reduced to 8.23 (0.05 mg/mL TSM group), 6.43 (0.5 mg/mL TSM group), and 5.47 (5 mg/mL TSM group), respectively. Next, we used Rh 123 staining to assess the level of MMP in PC12 cells. After 24 h exposure to 200 *μ*mol/L H_2_O_2_, the MMP was obviously reduced, as indicated by the decrease in Rh 123 fluorescence (Figure 8) and the MFI of Rh 123 quantified by FCM analysis (Figure 9) compared with nontreated control cells. When the cells cotreated with various concentrations of TSM (0.05 mg/mL, 0.5 mg/mL, and 5 mg/mL) and 200 *μ*mol/L H_2_O_2_ for 24 h revealed that the enhanced intensity of Rh 123 fluorescence compared to the H_2_O_2_-treated cells (Figure 9). These results implied that H_2_O_2_-induced dissipation of MMP is inhibited by TSM.

### 3.8. TSM Attenuates H_2_O_2_-Induced Increase in Intracellular Reactive Oxygen Species (ROS) Level

**Figure 10:**
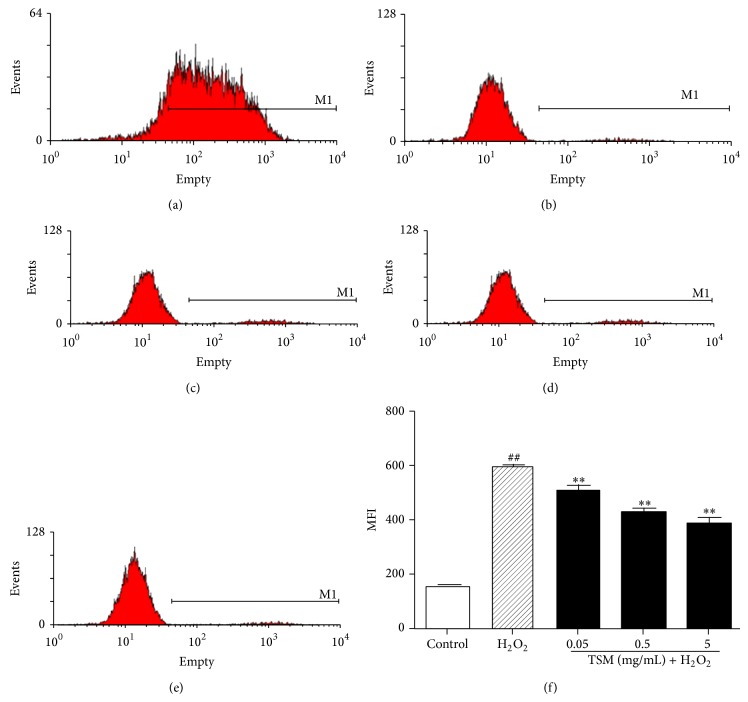
Revealing that TSM reduced H_2_O_2_-induced increase in intracellular ROS concentration in PC12 cells. The level of ROS was assessed by MFI of Dihydrorhodamine 123 (DHR) in ten thousand of PC12 cells. The control group did not change the ROS level but when cotreated with various concentrations of TSM would attenuate an increase in ROS level induced by H_2_O_2_ at 200 *μ*mol/L (TSM + H_2_O_2_). ^#^ *P* ≤ 0.05 and ^##^ *P* ≤ 0.01 as compared with the control group; ^*^ *P* ≤ 0.05 and ^**^ *P* ≤ 0.01 as compared with the H_2_O_2_ group.

As the cytotoxicity of H_2_O_2_ is mainly mediated by oxidative stress [50, 51], we investigated the effect Tong-Sai-Mai decoction on H_2_O_2_-induced ROS formation using DCFH-DA staining. Compared with nontreated control cells, the level of intracellular ROS was increased in PC12 cells treated with 200 *μ*mol/L H_2_O_2_ for 24 h, as indicated by the increase in DCF fluorescence (Figure 10) and the MFI of DCF quantified by the FCM analysis (Figure 10). But when the PC12 cells were cotreated with various concentrations of TSM (0.05 mg/mL, 0.5 mg/mL, and 5 mg/mL) and 200 *μ*mol/L H_2_O_2_ for 24 h, both of the DCF fluorescence (Figure 10) and the MFI of DCF (Figure 10) were significantly decreased, suggesting that H_2_O_2_-induced intracellular ROS accumulation is attenuated by TSM.

### 3.9. TSM Induces Overexpression of Bcl-2, Bax, and Caspase-3

**Figure 11:**
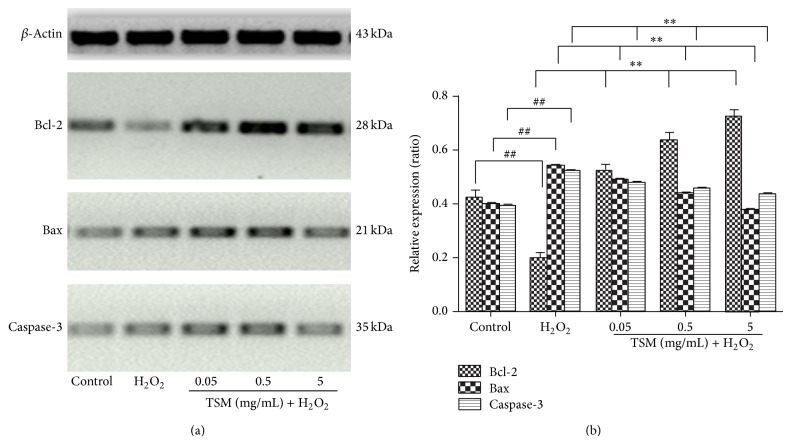
Showing that TSM induced change in bcl-2, bax, and caspase-3 proteins expressions in PC12 cells. The cells were treated with 200 *μ*mol/L H_2_O_2_ and 0.05, 0.5, and 5 mg/mL TSM in the presence of 200 *μ*mol/L H_2_O_2_. The protein expression was determined by Western blot assay. (b) Data were presented in mean *x* ± S.D. of results from the three independent experiments. ^#^ *P* ≤ 0.05 and ^##^ *P* ≤ 0.01 as compared with the control group; ^*^ *P* ≤ 0.05 and ^**^ *P* ≤ 0.01 as compared with the H_2_O_2_ group.

Bcl-2 is an antiapoptotic protein, bax is a proapoptotic protein, and caspase-3 is a frequently activated death protease, catalyzing the specific cleavage of many key cellular proteins. To explore whether TSM modulated the expressions of bcl-2, bax, and caspase-3 in PC12 cells and its response to the effect of H_2_O_2_ on bcl-2, bax, and caspase-3 expressions, the levels of bcl-2, bax, and caspase-3 were measured by Western blot analysis. As illustrated in Figure 11, 24 h treatment with various concentrations of TSM (0.05 mg/mL, 0.5 mg/mL, and 5 mg/mL) increased the amount of bcl-2 expression by 52.33%, 63.33%, and 72.33%, respectively. The exposure to 200 *μ*mol/L H_2_O_2_ for 24 h, on the other hand, reduced bcl-2 by 20%. However, the H_2_O_2_-induced decrease of bcl-2 was significantly abolished by cotreatment with various concentrations of TSM (0.05 mg/mL, 0.5 mg/mL, and 5 mg/mL) and 200 *μ*mol/L H_2_O_2_ for 24 h.

Apart from that, the exposure to 200 *μ*mol/L H_2_O_2_ for 24 h increased bax level by 54%. However, when cotreated with various concentrations of TSM (0.05 mg/mL, 0.5 mg/mL, and 5 mg/mL) and 200 *μ*mol/L H_2_O_2_ for 24 h, it would clearly reveal slight decrease in bax protein level expression with 49%, 43.67%, and 37.67%, respectively. Meanwhile, with the exposure to 200 *μ*mol/L H_2_O_2_ for 24 h, there was an increase in caspase-3 level by 52.33%. Therefore, when cotreated with various concentrations of TSM (0.05 mg/mL, 0.5 mg/mL, and 5 mg/mL) and 200 *μ*mol/L H_2_O_2_ for 24 h, it would obviously indicate a reduction in caspase-3 level expression by 48%, 45.33%, and 43.33%, respectively. These results revealed that TSM was able not only to promote the bcl-2 expression, but also to block the H_2_O_2_-induced downregulation of bcl-2 and decrease the bax and caspase-3 levels expressions.

### 3.10. TSM Antagonizes H_2_O_2_-Induced Overexpression of INOS

**Figure 12:**
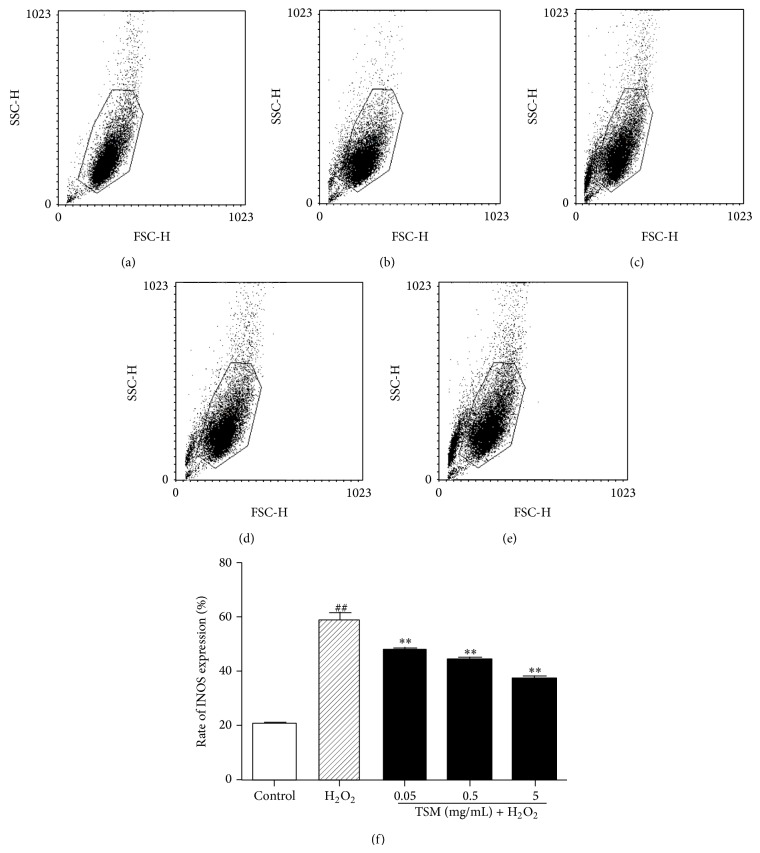
Influence of different treatments of TSM concentrations on the expression of INOS in PC12 cells. The rate of INOS expression was tested by FCM assay. (a) Control group. The cells were treated with 200 *μ*mol/L H_2_O_2_ (b) and 0.05, 0.5, and 5 mg/mL TSM in the presence of 200 *μ*mol/L H_2_O_2_ ((c), (d), and (e)). TSM could inhibit overexpression of INOS induced by 200 *μ*mol/L H_2_O_2_ (TSM + H_2_O_2_). ^#^ *P* ≤ 0.05 and ^##^ *P* ≤ 0.01 as compared with the control group; ^*^ *P* ≤ 0.05 and ^**^ *P* ≤ 0.01 as compared with the H_2_O_2_ group.

The FCM assay was applied to measure the expression of INOS. As shown in Figure 12, when PC12 cells were exposed to 200 *μ*mol/L for 24 h, the expression rate of INOS was obviously increased from 20.54% (control) to 58.83%. In contrast, while PC12 cells were cotreated with various concentrations of TSM (0.05 mg/mL, 0.5 mg/mL, and 5 mg/mL) and 200 *μ*mol/L H_2_O_2_ for 24 h, the upregulation of INOS expression was remarkably reduced to 47.34%, 43.79%, and 37.10%, respectively, suggesting that TSM inhibited not only the expression of INOS, but also the upregulation of INOS expression caused by H_2_O_2_ (Figure 12).

### 3.11. INOS-Specific Inhibitor Blocks H_2_O_2_-Induced Apoptosis

**Figure 13:**
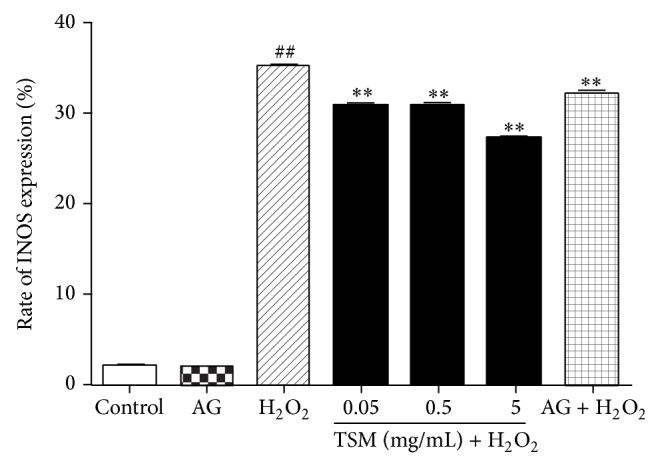
INOS-specific inhibitor blocked H_2_O_2_-induced apoptosis in PC12 cells. The cells were exposed to different treatments for 24 h, respectively. The percentage of PC12 cells apoptosis was assessed by the FCM assay. The cells were treated with 200 *μ*mol/L H_2_O_2_, 0.1 mmol/L AG, INOS-specific inhibitor, 0.1 mmol/L AG, INOS-specific inhibitor with 200 *μ*mol/L H_2_O_2_, and 0.05, 0.5, and 5 mg/mL TSM with 200 *μ*mol/L H_2_O_2_, indicating that AG can partly block the H_2_O_2_-induced PC12 cells apoptosis. ^#^ *P* ≤ 0.05 and ^##^ *P* ≤ 0.01 as compared with the control group; ^*^ *P* ≤ 0.05 and ^**^ *P* ≤ 0.01 as compared with the H_2_O_2_ group.

To further explore whether INOS was involved in H_2_O_2_-induced apoptosis, PC12 cells were cotreated with 200 *μ*mol/L H_2_O_2_ and the INOS-specific inhibitor, aminoguanidine (AG), at 0.1 mmol/L for 24 h. As indicated in Figure 13, 0.1 mmol/L AG alone did not induce the PC12 cells apoptosis, but the cotreatment with 0.1 mmol/L AG and 200 *μ*mol/L H_2_O_2_ partly blocked the apoptosis induced by H_2_O_2_, suggesting that INOS mediated H_2_O_2_-induced apoptosis in PC12 cells.

## 4. Discussion

TSM is a Chinese polyherbal formulation that widely applied in all the clinical studies such as in the treatment of thromboangiitis obliterans, arteriosclerotic occlusive disease, thrombophlebitis, deep venous thrombosis, brain stroke, and others. This formulation consists of 7 herbs such as* Astragalus membranaceus*,* Angelica sinensis*, Radix Achyranthis Bidentatae, honeysuckle,* Dendrobium nobile*, liquiritia glycyrrhiza, and Radix Scrophulariae. For example, there are research advances illustrating the usage of traditional Chinese medicine for neuroprotection in glaucoma [52].

Recently, Sen and Bian [53] reported that TSM provided the antisclerosis function against atherosclerosis through its antioxidant activity. But whether TSM afforded the neuroprotection in H_2_O_2_-induced cerebral ischemia disease model is unclear. In order to explore this phenomenon, we first investigated the H_2_O_2_ effects on PC12 cells in which retain dopaminergic characteristics. In the present study, we found that H_2_O_2_ caused a significant decrease in the cell viability and increase in the LDH leakage, confirming its neurotoxicity in PC12 cells. After the cells were cotreated with TSM, we discovered a significant increase in the cell viability and the decrease in the LDH leakage.

[Ca^2+^] plays a prominent role in the development of neurons and [Ca^2+^] overloading may result in the neuronal injury [54, 55]. In the acute insults such as hypoxic ischemia, it has been proposed that the resultant cell fate (necrosis, apoptosis, or survival) is dependent on the intracellular “Ca^2+^ setpoint” [56]. Accumulation evidence indicates that the cell injury, induced by modification of the redox state, is at least in part due to [Ca^2+^] dyshomeostasis via production of bioactive molecules such as ROS and RNS. From the study results, we found that TSM significantly reversed the H_2_O_2_-enhanced [Ca^2+^] concentration.

The DNA fragmentation and chromatin condensation are considered hallmark features of apoptotic processes. In the present study, TSM could significantly attenuate the DNA fragmentation expression. Furthermore, in the recent years, the cell apoptosis is closely related to the cell cycle [57]. This cell cycle can block the cell proliferation cycle and inducing apoptosis, and the apoptosis is normally accompanied by the cell cycle arrest. In the present study, we found that TSM could alleviate H_2_O_2_ on PC12 cells in G0/G1 phase to S phase.

There are some literatures demonstrating that ROS, such as superoxide (O^2−^), H_2_O_2_, and hydroxyl radical (OH^−^), are generated during the ischemia/reperfusion and have toxic effects in ischemic/reperfused myocardium [58]. In reality, there are a lot of Ca^2+^ regulatory proteins that can be influenced by ROS such as Na^+^/K^+^ ATPase [59]. ROS generation is considered to be one of the key signals for oxidative stress induced apoptosis [60]. Recent reports have shown that ROS can affect the mitochondrial function through the mitochondrial ATP-sensitive potassium (mito-KATP) channels and the mitochondrial permeability transition pore (Mptp) [24]. The irreversible opening of the Mptp has been thought to be involved in the lethal cell insult event like apoptosis [24]. In the present study, we found that Tong-Sai-Mai decoction (TSM) could attenuate the H_2_O_2_-induced increase in intracellular ROS level.

Apart from that, the MMP maintenance has proved its importance for ATP production and preservation of cellular homeostasis. For instance, Jones et al. reported that the oxidative stress by H_2_O_2_ exposure led to the MMP loss [61]. In the present study, we found that H_2_O_2_ induced the reduction of MMP thus inducing the dissipation of the MMP. TSM could significantly increase the cells dissipation of the MMP.

**Figure 14:**
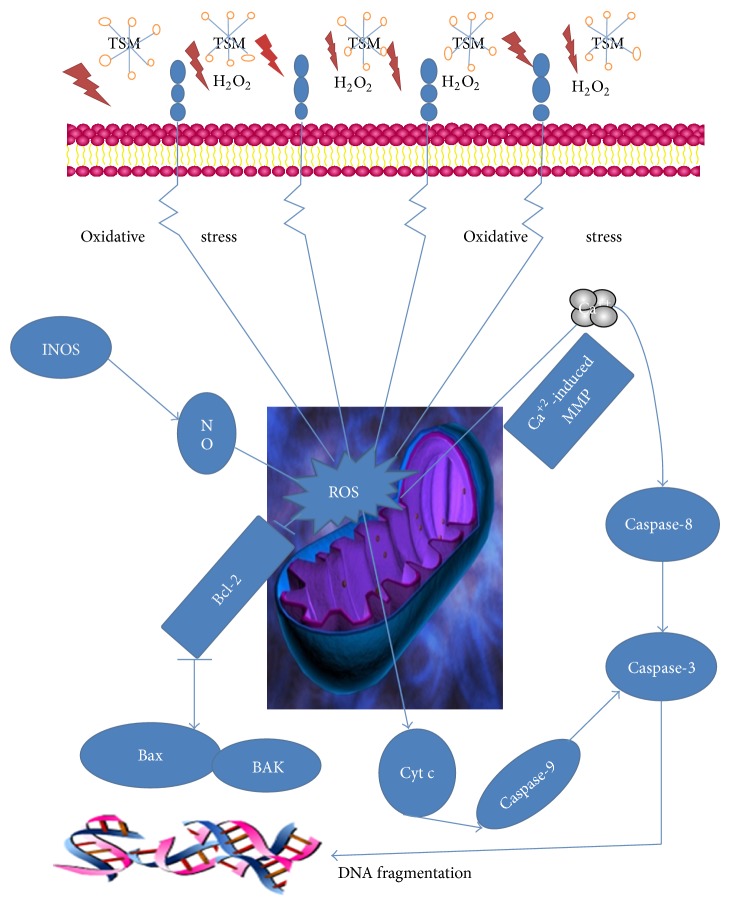
Proposed mechanisms for protecting H_2_O_2_-induced apoptosis of TSM in PC12 cells by bcl-2-mitochondria-ROS-INOS pathway.

There was an accumulating report illustrating homeopathic mother tincture of* Phytolacca decandra* [62] inducing apoptosis in skin melanoma cells by activating caspase-mediated signaling via reactive oxygen species elevation. More importantly, we found that for our lab first time that TSM could prevent H_2_O_2_-induced apoptosis in PC12 cells through the bcl-2-mitochondria-ROS-INOS pathway (Figure 14). TSM not only induced the bcl-2 overexpression, but also interdicted a decrease in bcl-2 expression induced by H_2_O_2_, implying that bcl-2 played the essential role in the neuroprotection of TSM against H_2_O_2_-induced apoptosis. Undoubtedly, bcl-2 has been proposed to prevent apoptosis by regulating the antioxidant pathway [63]. With this information, bcl-2 reduced the reactive oxygen intermediates (ROIs) intracellular concentrations that related to apoptosis [64]. The overexpression of bcl-2 could also increase the MMP stability [65] possibly by preventing mitochondria pore opening [66]. This finding provided an appropriate explanation for our data that TSM could block the H_2_O_2_-induced fall in MMP by inducing the bcl-2 overexpression and antagonizing the downregulation of bcl-2 overexpression.

Apart from that, bcl-2 might play an essential role in contributing the TSM inhibition on the INOS overexpression induced by H_2_O_2_. INOS is a main enzyme responsible for a large amount of NO production. The excessive NO produced by INOS can induce the neuronal apoptosis via the disruption of the mitochondrial membrane potential and activation of caspase-3 [67]. In the present study, we also found that TSM attenuated INOS expression and that INOS inhibitor AG partly protected PC12 cells against H_2_O_2_-induced apoptosis, indicating involvement of the INOS activity inhibition in the neuroprotection of TSM.

On the other hand, TSM could also reduce the bax and caspase-3 expressions. The increase in neuronal bax expression was described in the hippocampic and cerebellar neurons in the rat after the global ischemia [68], suggesting that bax plays role in the neuronal apoptosis [68]. Caspases are family of cysteine proteases which play crucial roles in the complex biochemical signaling events in triggering apoptosis [69]. Caspase-3 was believed to be a frequently activated death protease catalyzing the specific cleavage of many key cellular proteins [70]. In the current study, the bax and caspase-3 expressions in PC12 cells were significantly enhanced by H_2_O_2_ but significantly decreased by TSM. These results suggested that the neuroprotective effects of TSM in PC12 cells might be mediated, at least in part, by the inhibition of [Ca^2+^] overloading, bax, and caspase-3 expressions.

## 5. Conclusion

In conclusion, TSM afforded significant cytoprotection against H_2_O_2_-induced apoptosis. The cytoprotection caused by TSM might be contributed to attenuate the increase in LDH activity level, intracellular [Ca^2+^] concentration, and intracellular ROS levels but also could reverse the loss of MMP, blocking the INOS expression and reducing the bax expression. The antioxidant characteristics of TSM might be one of the main mechanisms involved in its cytoprotection against PC12 cells apoptosis induced by H_2_O_2_. Taken together, these findings suggested that overexpression of bcl-2 might play an essential role in the TSM cytoprotection induced by H_2_O_2_ in PC12 cells.
